# Preliminary Study of the Impact of Zwitterionic Ion-Exchange Resins on the Phenolic and Volatile Profiles of Fetească Neagră and Cabernet Sauvignon Wines

**DOI:** 10.3390/foods15101621

**Published:** 2026-05-07

**Authors:** Georgiana-Diana Gabur, Marcela Mihai, Marius-Mihai Zaharia, Fernando Sánchez-Suárez, Raquel Muñoz-Castells, Rafael A. Peinado, Carmen Teodosiu, Ionel-Bogdan Cioroiu, Iulian Gabur

**Affiliations:** 1 Faculty of Horticulture, “Ion Ionescu de la Brad” Iasi University of Life Sciences, 3 Mihail Sadoveanu Alley, 700490 Iasi, Romania; diana.gabur@iuls.ro; 2 Petru Poni Institute of Macromolecular Chemistry, 41A Grigore Ghica Voda Alley, 700487 Iasi, Romania; marcela.mihai@icmpp.ro (M.M.); zaharia.marius@icmpp.ro (M.-M.Z.); 3 Agricultural Chemistry, Soil Science and Microbiology Department, University of Córdoba, Campus of Rabanales, N-IV Road, Km 396, 14071 Córdoba, Spain; g62sasuf@uco.es (F.S.-S.); b52mucar@uco.es (R.M.-C.); qe1peamr@uco.es (R.A.P.); 4 “Cristofor Simionescu” Faculty of Chemical, Engineering and Environmental Protection, “Gheorghe Asachi” Technical University of Iasi, 73 Prof. D. Mangeron Blvd., 700050 Iasi, Romania; 5 Romanian Academy—Iasi Branch, Research Centre for Oenology, 9H Mihail Sadoveanu Alley, 700490 Iasi, Romania; bogdan.cioroiu@acadiasi.ro

**Keywords:** zwitterionic resin, volatile aroma compounds, phenolic compounds, Cabernet Sauvignon, Fetească neagră, statistical analysis

## Abstract

Zwitterionic ion-exchange polymers, known for advanced sorption properties, were used in this study to investigate their impact on the phenolic and volatile aroma compounds of Fetească neagră (FN) and Cabernet Sauvignon (CS) wines. Treatments with these compounds showed decreases in total phenolic content from 7.08 to 5.79 g/L gallic acid equivalents (GAE) in CS and from 5.96 to 4.76 g/L GAE in FN, reducing tannins and reactive phenolics and enhancing colloidal stability. Analysis of volatile compounds revealed selective increases in key esters and higher alcohols, contributing to enhanced fruity notes reaching 22.9% in CS and 26.8% in FN, and floral notes reaching 14.9% in CS and 20.4% in FN. Principal component analysis (PCA) showed clear separation by variety (PC1: 54.8%) and treatment (PC2: 30.1%), while heatmap clustering highlighted variety-specific volatile profiles. These results demonstrate that zwitterionic resin treatment represents a novel approach for controlled modulation of wine composition and aroma, enhancing stability and aromatic complexity while preserving varietal typicity.

## 1. Introduction

Red wines are highly complex matrices composed of numerous volatile and non-volatile compounds that contribute to their sensory, chemical, and health-related properties. This complexity arises from the interplay of grape composition, fermentation dynamics, aging processes, and post-fermentation treatments, all of which shape the aromatic and phenolic profiles that define wine quality [1,2]. Among post-fermentation strategies, ion-exchange treatments have been explored for their capacity to adjust pH, stabilize tartaric salts, and modulate certain chemical components. While conventional cationic and anionic resins have demonstrated efficacy in these applications, their effects on the fine balance of aroma and phenolic composition remain limited. Zwitterionic ion-exchange resins possess a dual-charged mechanism enabling selective interactions with both anionic and cationic species, potentially influencing volatile compounds, phenolics, and overall aroma expression more precisely. Ion-exchange technology is widely applied in oenology for tartrate stabilization and acidification [3,4]. Within the European Union, the application of ion-exchange resins in winemaking is strictly regulated. In current oenological practice, cation-exchange resins operating in the acid cycle (H^+^ form) represent the only type authorized for wine treatment under Commission Delegated Regulation (EU) 2019/934, which supplements Regulation (EU) No 1308/2013 [5]. According to the International Organisation of Vine and Wine (OIV) International Code of Oenological Practices [6], these treatments must employ resins regenerated in the acid cycle and be applied primarily for the removal of excess cations. The cumulative increase in titratable acidity must not exceed 54 meq/L (approximately 4 g/L expressed as tartaric acid), and the pH of the treated wine should not fall below 3.0. In addition, excessive removal of metallic cations that could negatively affect wine composition and sensory properties must be avoided.

Conventional cation-exchange resins are commonly used to reduce cation concentrations, decrease pH, and improve tartrate stability [3]. Tartrate precipitation remains one of the main technological challenges in winemaking, and several stabilization strategies have been developed, including cold stabilization, electrodialysis, protective colloids, and ion-exchange treatments. Despite their effectiveness, conventional cation-exchange resins may also cause excessive acidification and non-selective removal of certain wine constituents. Previous studies have reported reductions in total phenolic content associated with hydrophobic interactions and hydrogen bonding between phenolic compounds and resin matrices, which may lead to partial losses of pigments and tannins [4]. Because phenolic compounds play a central role in determining color stability, mouthfeel, and aging potential, stabilization strategies must be carefully evaluated to preserve these key quality attributes.

In recent years, zwitterionic ion-exchange resins have emerged as a new class of functional materials. These resins contain both positively and negatively charged functional groups within the same polymeric matrix, enabling simultaneous interactions with cations and anions. This dual-charged structure may allow more selective modifications of chemical composition compared with conventional ion-exchange systems. Consequently, such materials could potentially influence the balance between organic acids, phenolic compounds, and volatile constituents, thereby affecting the overall aroma profile and compositional stability of wine. However, studies investigating the application of zwitterionic resins in oenological systems remain extremely limited.

According to our knowledge, no previous studies have explored the use of zwitterionic ion-exchange resin, specifically functionalized with ethylenediamine (EDA), for modulating the phenolic and volatile composition of wine. Therefore, this study aimed to evaluate the impact of zwitterionic resin treatment on the phenolic profile, volatile compounds, and aromatic series of red wines, providing new insights into the potential application of this emerging technology in winemaking.

## 2. Materials and Methods

### 2.1. Synthesis of Zwitterionic Resin Beads and Winemaking Process

**Figure 1:**
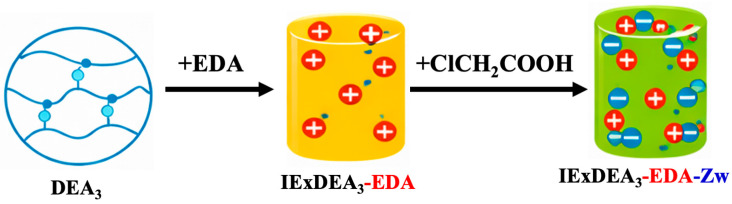
Synthesis pathway of cationic and zwitterionic ionic-exchange resins derived from DEA_3_ acrylic copolymer.

The zwitterionic resin beads were prepared in accordance with our previously published method [7] via the polymer-analogous modification of an acrylic copolymer (3% divinyl benzene, 77% ethyl acrylate, 20% acrylonitrile) (DEA_3_). The methodology for synthesis involved two sequential steps. The first step was an aminolysis reaction of the copolymer with ethylenediamine (EDA), thereby creating the respective cationic resins (IExDEA_3_-EDA). The second step involved carboxymethylation of the resulting intermediates with sodium chloroacetate to yield the zwitterionic functionalized resins (IExDEA_3_-EDA-Zw) as shown in Figure 1.

For activation of the functional groups, 10 mL of zwitterionic resin (IE-Zw) was treated with 20 mL of sodium hydroxide (NaOH) solution and allowed to stand for 1 h with periodic stirring to ensure homogeneity. The NaOH solution was then removed, and the IE-Zw was washed with distilled water until neutral pH was reached. Neutrality was monitored using phenolphthalein as an indicator and washing continued until the disappearance of the characteristic pink color.

The study was conducted using *Vitis vinifera* L. cv. *Cabernet Sauvignon* (CS) and *Fetească neagră* (FN) grapes, hand-harvested at optimal technological maturity from the experimental vineyards of the “Ion Ionescu de la Brad” Iasi University of Life Sciences (Iași, Romania). The winemaking process followed a standardized protocol at the university’s pilot-scale facility. After destemming and crushing, the must underwent maceration–fermentation in temperature-controlled stainless-steel tanks (20 °C). Alcoholic fermentation was conducted using commercial *Saccharomyces cerevisiae* (Xr Grand Rouge, Lamothe-Abiet; 0.25 g/L), selected for their capacity to preserve varietal aromatic precursors. Standard EU-compliant treatments included Lallzyme HC pectolytic enzyme (2 mL/ton) at crushing and potassium metabisulfite (40 mg/L total SO_2_) pre-fermentation, during alcoholic fermentation (10–12 days, 20 °C, to <4 g/L sugars). After fermentation completion, wines were stabilized with OptiEsters^TM^ (30 g/hL) and OptiThiols^TM^ (per manufacturer Lallemand Inc., Montreal, QC, Canada), filtered and bottled. To ensure compositional integrity prior to the experimental zwitterionic resin treatments, the bottled wines were stored horizontally in a dedicated cellar under controlled conditions (13.5 °C and constant 75% relative humidity) in total darkness, until further analysis.

**Figure 2:**
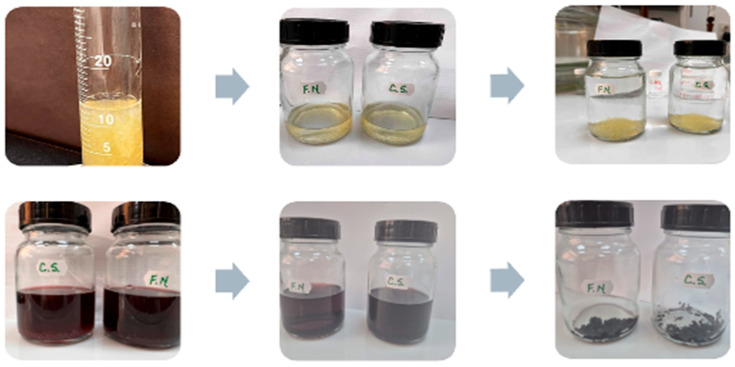
Activation of the zwitterionic ion-exchange resin (first line) and its application in red wine treatment (second line).

For the zwitterionic ion-exchange treatment, 100 mL of each wine sample was combined with 10 mL of the activated IE-Zw resin and agitated on an orbital shaker for 24 h to ensure adequate contact for ion-exchange and stabilization. Following the treatment, the wines were separated from the resin and stored at 12 °C until further analysis (Figure 2). All analyses were performed in triplicate (*n* = 3) per wine sample, with data reported as mean ± SD and statistical significance assessed via ANOVA and Tukey’s post hoc test (*p* < 0.05).

The figures represent samples illustrating the state of the zwitterionic resin during treatment and wine samples (CS and FN) before and after zwitterionic ion-exchange treatment for one representative replicate.

### 2.2. Identification and Quantification of Aroma Compounds

#### 2.2.1. Major Volatile Compounds

The profile of major volatile compounds in the wine samples was determined using gas chromatography coupled with a flame ionization detector (GC–FID). Analyses were carried out on an HP 6890 Gas Chromatograph equipped with an FID and fitted with a CP-WAX 57 CB Capillary Column (50 m length, 0.25 mm internal diameter, 0.4 μm film thickness). Identification and quantification of the compounds were performed using calibration curves established for each analyte according to previous research [8,9]. Chromatographic separation was achieved using a split injection mode with a split ratio of 30:1. The oven temperature program started at 50 °C and was maintained for 15 min, followed by a gradual increase at a rate of 4 °C min^−1^ until reaching 190 °C, where it was held for 2 min (total run time 52 min). The injector temperature was set at 270 °C, while the detector operated at 300 °C. Helium was used as the carrier gas. The initial flow rate was adjusted to 0.7 mL min^−1^ for the first 16 min, after which it was increased to 1.1 mL min^−1^ until the completion of the chromatographic run (52 min). Before chromatographic analysis, tartaric acid was removed from the wine matrix by precipitation using 0.2 g of calcium carbonate, followed by centrifugation at 300 g and 4 °C. For the analysis, 10 mL of wine sample was fortified with 1 mL of 4-methyl-2-pentanol solution (1 g L^−1^), used as an internal standard. Subsequently, 0.5 μL of the prepared sample was injected directly into the split/splitless injector of the GC instrument.

#### 2.2.2. Minor Volatile Compounds

##### Extraction of Minor Aroma Compounds

Minor aroma compounds were extracted using stir bar sorptive extraction (SBSE), following the procedure described by Lopez de Lerma et al. [10] and Dumitriu et al. [2]. Prior to extraction, the wine samples were diluted (1:10, v/v) with a hydroethanolic solution containing 12% ethanol. The solution was previously adjusted to pH 3.5 by adding 2.6 g L^−1^ tartaric acid and 2.2 g L^−1^ potassium bitartrate. A polydimethylsiloxane (PDMS)-coated stir bar (0.5 mm film thickness, 10 mm length; Gerstel GmbH, Mülheim an der Ruhr, Germany) was introduced into a 10 mL headspace glass vial containing 10 mL of the prepared sample. Subsequently, 0.1 mL of ethyl nonanoate solution (0.45 mg L^−1^) was added as an internal standard. The vial was sealed with a Teflon-lined crimp cap.

Extraction was carried out under stirring at 1500 rpm for 100 min at 25 °C. After completion of the extraction step, the stir bar was removed and placed in a glass thermal desorption tube for subsequent GC–MS analysis.

##### Determination of Minor Aroma

The thermal desorption tube containing the stir bar was introduced into a thermal desorption unit (Gerstel TDS 2) coupled to the GC-MS system. During the desorption process, the stir bar was heated in order to release the extracted compounds, which were subsequently transferred to a cooled injection system/programmed temperature vaporizer (CIS 4 PTV) equipped with a Tenax adsorption tube. Thermal desorption was carried out starting at 35 °C, followed by a rapid temperature increase at 120 °C min^−1^ up to 280 °C, where it was maintained for 10 min. Helium was used as the carrier gas with a flow rate of 3 mL min^−1^. The CIS injector temperature was initially maintained at 25 °C throughout the desorption period and then increased to 280 °C at a heating rate of 12 °C s^−1^ under splitless conditions, with a final holding time of 7 min.

Chromatographic separation was achieved using a capillary column (Agilent 19091S Capillary Column, 30 m × 0.25 mm i.d., 0.25 μm film thickness) from Agilent (Agilent Technologies, Santa Clara, CA, USA). Helium served as the carrier gas with a constant column flow rate of 1 mL min^−1^. The oven temperature program started at 50 °C and was maintained for 2 min, followed by a temperature increase of 4 °C min^−1^ to 190 °C, where it was held for 10 min.

The mass spectrometer operated in scan mode, monitoring ions within a mass range of 39–300 *m*/*z*. Identification of the volatile compounds was achieved by comparing retention times and mass spectra with those available in the Wiley Mass Spectral Library as well as with authentic reference standards. Quantification was performed using calibration curves constructed from standard solutions with known concentrations that were subjected to the same analytical procedure as the samples. Data processing and compound confirmation were carried out using the Hewlett-Packard ChemStation software version B.03.02 (Palo Alto, CA, USA).

### 2.3. Aroma Series

Aroma series represent groups of volatile compounds that exhibit similar sensory descriptors. The contribution of each series was estimated by calculating the sum of the odor activity values (OAVs) of the compounds included in that category. Since a single volatile compound may represent multiple sensory descriptors, it can be associated with more than one aroma series [11].

Based on the sensory characteristics reported in the literature, nine aroma series were considered in this study: fruity, green fruit, green, creamy, citrus, chemistry, honey, waxy and floral. In general, volatile compounds with an odor activity value (OAV) equal to or higher than 1 are regarded as odor-active and are therefore likely to contribute to the overall aroma perception of the sample.

### 2.4. Determination of Phenolics, Tannins, Anthocyanins and Color Parameters

Total phenolics were evaluated spectrophotometrically using the total polyphenol index (TPI) and the Folin–Ciocalteu assay. For TPI determination, wine samples were clarified by filtration or centrifugation, diluted with distilled water, and the absorbance was measured at 280 nm using a UV-Vis spectrophotometer (Analytik Jena Specord 200, GmbH+Co. KG, Jena, Germany) equipped with 1 cm quartz cuvettes. Total reducing phenolics were determined using the Folin–Ciocalteu method, based on the reduction in phosphomolybdotungstic complexes under alkaline conditions at 760 nm. Results were expressed as gallic acid equivalents (GAE).

Tannin content was estimated using an adapted protocol derived from the European Pharmacopoeia method for tannin determination (EP 2.8.14) [12] by replacing hide powder with PVPP as an alternative adsorbent due to its strong affinity for polymeric phenols. Wine samples were analyzed before and after PVPP treatment, and the decrease in phenolic content was attributed to tannin fractions.

Monomeric anthocyanins were determined using the pH differential method, which exploits the reversible structural transformation of anthocyanins between colored and colorless forms at different pH values. Absorbance was measured at 520 nm in buffered systems at pH 0.6 and pH 3.5.

pH, turbidity (Lovibond TB300 IR, NTU), and electrical conductivity (μS/cm) were determined at 20 °C.

Wine color was characterized using the CIELab color system, determining the parameters L* (lightness), a* (green–red coordinate), and b* (blue–yellow coordinate). Additional chromatic descriptors, including chroma, hue angle and total color difference, were calculated to evaluate color intensity and perceptible differences among samples.

### 2.5. Statistical Treatment

Statistical analyses were conducted to evaluate differences between wines treated with the zwitterionic ion-exchange resin and initial samples. Multivariate analyses, including principal component analysis (PCA) and heatmaps, were performed in R code (v. 4.5.3), packages “tidyverse”,”rstatix” and “multcompView”, to visualize patterns, relationships, and clustering among treatments. All analyses were based on replicate measurements, and statistical significance was considered at a 95% confidence level.

## 3. Results and Discussion

### 3.1. Impact of Zwitterionic Resin on Physical–Chemical Parameters

**Figure 3:**
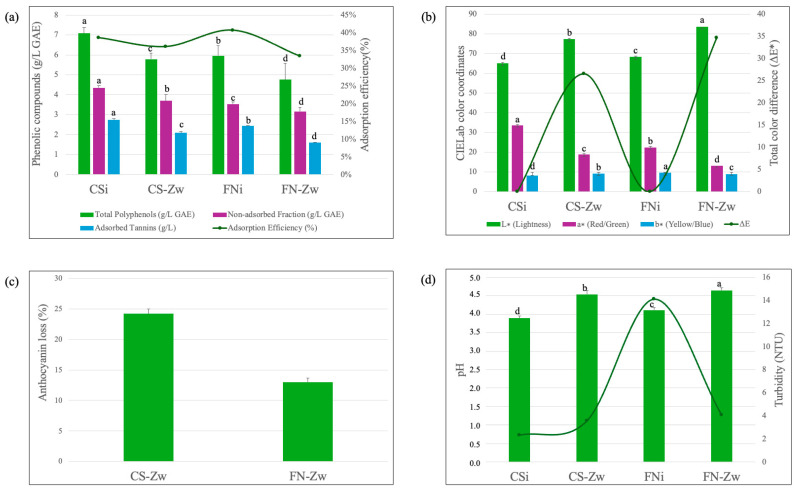
Impact of the zwitterionic ion-exchange resin on: (**a**) total polyphenolic index (TPI) adsorption and efficiency of the zwitterionic resin treatment; (**b**) wine chromatic characteristics; (**c**) anthocyanin stability; (**d**) pH and turbidity; different letters indicate significant differences (ANOVA and Tukey’s HSD, *p* < 0.05).

Treatment with zwitterionic ion-exchange resins led to a reduction in the total polyphenol index (TPI) of both wine varieties (Figure 3a).

In CS, TPI decreased from 7.08 to 5.79 g/L GAE, while in FN this decreased from 5.96 to 4.76 g/L GAE, confirming the resin’s capacity to effectively sequester phenolic compounds. The concomitant reduction in tannin fractions aligns with the subtractive mechanism characteristic of ion-exchange processes, as previously described by Martínez-Pérez et al. [13].

This behavior highlights the resin’s role in selectively removing reactive phenolic species linked to colloidal instability. Adsorption efficiency was higher in FN (40.6%) than CS (38.4%), indicating greater affinity of the zwitterionic matrix for FN’s phenolic profile. This selectivity likely arises from hydrophobic interactions and hydrogen bonding between the resin backbone and polyphenol hydroxyls. Such targeted removal should reduce precipitation risk, enhancing colloidal stability and shelf-life. Unlike previous ion-exchange studies reporting only minor tannin reductions [14,15], our treatment achieved substantial phenolic sequestration.

Colorimetric analysis revealed significant clarification following zwitterionic resin treatment (Figure 3b). Lightness (L*) increased markedly in both grape varieties, with FN reaching 83.58 vs. 77.39 in CS, indicating superior transparency in FN. The a* (redness) parameter decreased more pronouncedly in CS, yielding higher total color difference (ΔE*) in FN (34.74) than CS (26.59). Despite anthocyanin reduction, Martínez-Pérez et al. [13] confirm that ion-exchange treated wines maintain chromatic stability during storage, while Traynor et al. [16] attribute such shifts to changes in phenolic composition and retention of phenolic compounds by the ion-exchange resin.

To elucidate these chromatic changes, monomeric anthocyanin behavior was examined. As shown in Figure 3c, the zwitterionic resin exhibited selectivity toward monomeric anthocyanins, with lower relative loss in FN (13.0%) than CS (24.2%). This varietal difference suggests FN’s native anthocyanin profile resists resin interaction, likely due to higher methoxylation or stronger copigmentation protecting against adsorption. Martínez-Pérez et al. reported similar initial pigment reductions followed by improved storage stability, while Pismenskaya et al. [17] show that ion-exchange efficiency depends on the structural and ionization properties of anthocyanins, which vary with grape variety.

The decrease in total anthocyanin content is likely due to adsorption of flavylium cations onto the resin surface at wine pH [4]. Comparable reductions have been reported in wines treated with cation-exchange resins [14,15,18]. This effect may be explained by the predominance of anthocyanins in the flavylium cation form at wine pH values, particularly under more acidic conditions [19]. As positively charged species, flavylium ions can be preferentially adsorbed onto the resin and exchanged with hydrogen ions (H^+^), similarly to other cations present in wine. A comparable mechanism was proposed by Mislata et al. [18] to account for anthocyanin losses observed after resin treatment of Tempranillo wines. These findings confirm that pigment modulation occurs in a grape variety- dependent manner, allowing the partial preservation of chromatic identity, particularly in FN wines.

Interestingly, the zwitterionic resin treatment led to an increase in wine pH, from 4.11 to 4.65 for FN and from 3.89 to 4.54 in CS (Figure 3d), in contrast to classical cation-exchange treatments that typically lower pH [15]. This behavior can be attributed to the dual charged nature of the zwitterionic matrix, which allows selective adsorption of both organic acid anions and hydrated cations. By reducing the concentration of free H^+^ ions in solution, the treatment increases pH while simultaneously modifying the ionic environment of the wine. While this pH increase demonstrates the efficiency of zwitterionic resins in modulating wine ions, it may affect wine perception by reducing perceived acidity, altering color stability in red wines, and slightly increasing susceptibility to microbial spoilage. Therefore, monitoring pH after treatment is recommended to ensure wine quality is maintained.

The efficiency of stabilization was corroborated by a marked reduction in turbidity, particularly in FN, where values decreased from 14.10 NTU to 4.06 NTU, reflecting a highly effective clarification process. This outcome aligns with OIV objectives aimed at ensuring physicochemical stability while preserving the intrinsic characteristics of the wine. Furthermore, the observed increase in electrical conductivity, from 2788 to 3867 μS/cm, confirms the occurrence of intense ion-exchange interactions between the wine matrix and the resin. Such conductivity changes reflect modifications in the ionic balance of the system and are consistent with the mechanistic framework proposed by Mira et al. [4]. Martínez-Pérez et al. [13] also demonstrated that ion-exchange treatments can surpass additive-based stabilizers, such as carboxymethyl cellulose, in terms of stabilization efficiency. In red wines, variations in electrical conductivity following induced tartrate crystallization are commonly used to assess tartaric stability, as described by Bosso et al. [20]. Conductivity changes greater than 100 μS/cm after crystallization are considered to indicate adequate tartaric stability [20].

Zwitterionic resin treatment enhances clarity and physicochemical stability while preserving varietal characteristics. This aligns with Martínez Moreno et al. [19], who showed cation-exchange resins improve pH adjustment and tartrate stabilization in Monastrell wines. Overall, zwitterionic resins offer a promising multifunctional approach for integrated ionic regulation and stabilization, enhancing red wine quality.

### 3.2. Volatile Compounds

In this study, eight volatile families, alcohols, esters, aldehydes, ketones, furanic compounds, lactones, terpenes, and norisoprenoids were evaluated to investigate the effect of zwitterionic ion-exchange resin treatment on the volatile profile of red wines. Treatment of CS and FN wines produced selective enhancement of aroma intensity. A total of 57 compounds were quantified (12 alcohols, 19 esters, 9 aldehydes, 3 ketones, 3 furanic compounds, 3 lactones, 8 terpenes/norisoprenoids) (Table 1).

#### 3.2.1. Alcohols

Higher alcohols (fusel alcohols), primarily arising from yeast metabolism via amino acid catabolism (Ehrlich pathway), represent the most abundant volatile fraction in the studied wines. In CS, total major alcohols increased from 653 mg/L in the initial sample to 728 mg/L in the treated sample, reflecting significant increases in key aroma-active compounds such as 3-methylbutanol (from 300 to 333 mg/L) and 2-phenylethanol (from 58 to 68 mg/L). Similarly, in FN, which had lower initial concentrations, 3-methylbutanol and 2-phenylethanol increased from 186 to 211 mg/L and from 36 to 43 mg/L, respectively (Table 1). These alcohols are key contributors to floral and fruity aromas at moderate concentrations, with 2-phenylethanol providing characteristic rose-like notes [21]; however, higher concentrations may impart unpleasant solvent-like notes.

Minor alcohols also exhibited significant increases following the zwitterionic resin treatment. For instance, hexanol increased from 483 to 615 μg/L in CS and from 317 to 396 μg/L in FN, contributing green and fresh odor notes [22]. Octanol similarly increased, contributing to subtle fruity and floral layers, while 2-furanmethanol, responsible for caramel-like and sweet notes, exhibited substantial increases of 95.2% in CS and 32.7% in FN. These results indicate that the zwitterionic resin releases previously matrix-bound higher alcohols, thereby increasing their direct injection GC-FID detectability while enhancing minor alcohol headspace partitioning (SBSE-GC-MS) [15,23].

This phenomenon, often described as “matrix refinement”, aligns with the observed increases in both major and minor alcohols. Zwitterionic ion-exchange resins contain both positively and negatively charged functional groups within a single polymeric matrix, enabling more complex interactions with wine constituents compared with conventional cation-exchange resins. Such interactions can modify the non-volatile matrix environment, thereby influencing how volatile compounds partition between the liquid phase and the headspace and ultimately affecting their analytical detectability. The influence of the non-volatile wine matrix on the volatility and headspace partitioning of aroma compounds has been widely reported, as subtle matrix modifications can either enhance or suppress the apparent concentrations of volatile alcohols and other aroma-active molecules during analysis. These effects suggest that both the selective ion-exchange properties of the zwitterionic resin and the resulting modifications of the wine matrix synergistically contribute to the elevated levels of volatile alcohols observed in treated samples [24].

The observed increases in isoamyl alcohol and isobutanol further supports a role for amino acid catabolism transformation, potentially induced by subtle abiotic stress or ionic reorganization within the wine matrix [9]. These changes, together with the increases in both major and minor alcohols, contribute significantly to the complexity and intensity of the varietal aroma, particularly enhancing floral and fruity notes. The results demonstrate that zwitterionic ion-exchange treatment not only preserves volatile alcohols but also enhances their partitioning into the headspace, thereby increasing the sensory impact of fermentative alcohols in both Cabernet Sauvignon and Fetească neagră wines.

#### 3.2.2. Esters

Esters, formed enzymatically during alcoholic fermentation via condensation of alcohols and acyl-CoA intermediates [25], represent the primary contributors to fruity and floral notes. In both grape varieties, zwitterionic treatment resulted in clear increases in major esters, including ethyl acetate, ethyl lactate, and diethyl succinate, as well as in minor esters with high odor impact. For example, isoamyl acetate, a compound imparting banana-like aroma, increased markedly in FN from 160 to 277 mg/L, while 2-phenylethanol acetate, associated with floral and honey notes, rose from 234 to 296 mg/L (Table 1). Other important esters, such as ethyl octanoate, ethyl butanoate and ethyl 3-methylbutanoate, also exhibited substantial increases contributing additional fruity, floral and pear notes [2].

These results suggest that the zwitterionic resin release matrix-bound esters through selective ion removal and pH changes (from 4.11 to 4.65), increasing their total concentration and minor ester headspace availability, as detected by GC-FID and SBSE-GC-MS [18]. The observed increases in both major and minor esters indicate that the resin’s 3% cross-linked matrix prevents adsorption of these non-polar molecules due to steric exclusion, preserving and amplifying the fruity and floral aromatic profile. This behavior is consistent with previous observations that esters represent the most abundant and impactful aroma compounds after higher alcohols, playing a crucial role in the sensory complexity and varietal expression of wines [22].

Notably, ethyl hexanoate exhibited grape variety-dependent behavior, decreasing by 63.6% in Cabernet Sauvingon but remaining relatively stable in Fetească neagră. This suggests selective matrix interactions that do not compromise the overall fruity enhancement driven by other esters. Additionally, 4-methylbenzaldehyde exhibited substantial increases in both grape varieties, with rises of 108% in Cabernet Sauvignon and 185% in Fetească neagră, indicating efficient de-complexation from polyphenolic matrices.

Previous studies on cationic resin treatments have reported that only a limited number of fruity esters exceed their odor thresholds, while many other volatile compounds decrease [3,15,26]. In contrast, the present study demonstrates that zwitterionic resin treatment can selectively enhance the headspace availability of higher alcohols and esters without negatively affecting sensitive aldehydes and ketones. This highlights the unique dual-charged mechanism of zwitterionic resins, which allows for fine-tuning of wine aroma and phenolic interactions in a manner not observed with conventional ion-exchange treatments.

#### 3.2.3. Aldehydes and Ketones

Aldehydes and ketones represent key fermentation-derived and secondary aroma compounds in wine, though their concentrations are typically lower than those of alcohols and esters. In this study, the zwitterionic resin treatment selectively affected these volatile classes, exhibiting distinct behavior between major and minor comppunds. Aldehydes such as acetaldehyde remained largely stable after treatment, with concentrations of 51–53 mg/L in CS and 111–112 mg/L in FN, indicating that the zwitterionic process does not induce oxidative reactions or unwanted chemical modifications. This stability confirms the mild, non-destructive nature of the treatment, consistent with previous reports that aldehydes often exhibit limited responssiveness to post-fermentation chemical perturbations [27].

In contrast, minor aldehydes exhibited more pronounced changes, particularly 4-methylbenzaldehyde in CS, which increased 2.1-fold from 49 to 102 mg/L. This likely results from a de-complexation mechanism, where the zwitterionic functional groups disrupt weak interactions between aldehydes and polyphenolic fractions, releasing the free, aroma-active forms [28]. Other minor aldehydes, such as octanal, nonanal, and phenylacetaldehyde, showed minor fluctuations, suggesting selective liberation depending on the compound’s affinity for the wine matrix.

Ketones demonstrated relative stability in response to treatment. Major ketones, including acetoin, showed minimal changes, while minor ketones such as 3-heptanone and benzophenone exhibited only slight variations. The stability of aldehydes and ketones contrasts with the pronounced increases in fermentative alcohols and esters, highlighting that zwitterionic resins act selectively on fermentation-related compounds rather than on volatiles derived from aging or secondary processes.

#### 3.2.4. Furanic Compounds and Lactones

Furanic compounds and lactones, derived from grape sugars and fermentation reactions (pentose dehydration, Maillard reactions), showed substantial increases following zwitterionic resin treatment. In Cabernet Sauvignon, furfural was higher, reaching 473 μg/L compared with 355 μg/L initially, while in Fetească neagră it reached 489 μg/L from 361 μg/L. Similarly, butyrolactone concentrations in CS reached approximately 49,198 μg/L from 35,923 μg/L (Table 1).

These pronounced changes reflect de-complexation, whereby zwitterionic functional groups disrupt weak interactions between furanic compounds or lactones and polyphenolic/metallic components. Specifically, metal ions stabilizing polyphenol–lactone complexes are removed, freeing bound molecules into the volatile fraction [29]. This selective liberation is particularly relevant in high-polyphenol matrices like FN, where aroma actives often remain masked.

The increases in furanic compounds and lactones contribute to enhanced sensory attributes, including creamy, caramel, and roasted notes, thereby enriching the wine’s aromatic complexity and depth. The effect complements the increases observed in fermentative alcohols and esters, highlighting that zwitterionic treatment can simultaneously modulate multiple aromatic families through matrix modification without inducing unwanted chemical reactions. These findings position zwitterionic resins as a tool for refining the aroma profile and improving the sensory perception of high-quality red wines.

#### 3.2.5. Terpenes and Norisoprenoids

Terpenes and norisoprenoids are key varietal aroma compounds, derived from the grape, and are often associated with floral and notes (e.g., rose, citrus). Despite their typically low concentrations, these compounds can have a significant sensory impact due to their low odor detection thresholds.

In this study, β-damascenone increased from 2.1 to 3 μg/L in CS and 2.2 to 2.9 μg/L in FN. This suggests zwitterionic treatment either prevents loss of sensitive volatiles or facilitates mild hydrolysis of glycosidically bound precursors, enhancing free aroma-active forms. These changes reinforce varietal typicity in both varieties.

Other terpenes, including β-citronellol, citral, and nerolidol, exhibited only minor fluctuations after treatment, confirming that the resin selectively influences compounds sensitive to the ionic environment while largely preserving the integrity of grape-derived volatiles. This selective effect highlights the precision of zwitterionic treatment, which can modulate the expression of highly odor-active molecules without disrupting the broader varietal aroma profile.

Mislata et al. (2021) [18] showed ion-exchange treatment preserves Tempranillo aromatic profiles even after prolonged oak aging, with 20% treated wines exhibiting no significant aroma changes. Similarly, zwitterionic treatment enhances key varietal notes by increasing specific norisoprenoids while stabilizing sensitive terpenes, yielding more expressive, complex aromatic profiles.

The combined findings demonstrate zwitterionic resin treatment functions as a matrix-refining agent rather than simple stripping or deacidification. The treatment increases the headspace availability of higher alcohols and esters, improving the perception of fruity and floral aromas, and facilitates the de-complexation of aldehydes, furanic compounds, and lactones from polyphenolic matrices, resulting in higher detectable concentrations and enhanced aroma intensity. Additionally, the treatment preserves and amplifies varietal markers, including terpenes and norisoprenoids, enhancing aromatic complexity without oxidative degradation. Zwitterionic treatment represents a selective, mild strategy for post-fermentation aroma optimization in high-quality varieties like CS and FN.

### 3.3. Aromatic Series

Nine aroma series were evaluated in CS and FN wines: fruity, green fruit, green, creamy, citrus, chemical, honey, waxy, and floral. These series were quantified to provide an integrative view of the aroma profile and to assess the impact of zwitterionic resin treatment.

**Figure 4:**
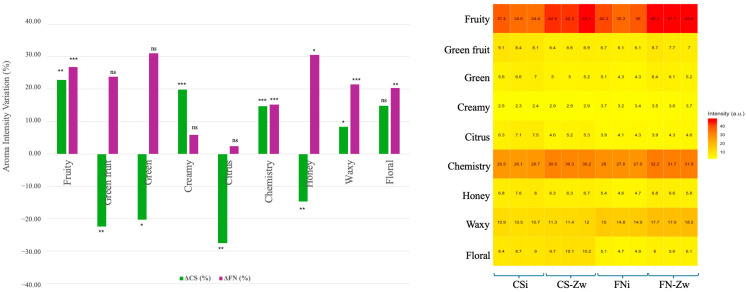
Impact of zwitterionic resin treatment on the aromatic profile of Cabernet Sauvignon (CS) and Fetească neagră (FN) wines: (**left**) aroma intensity variation (%), where asterisks indicate statistical significance (* *p* < 0.05, ** *p* < 0.01, *** *p* < 0.001, ns: non-significant); (**right**) heatmap representing absolute aroma series intensity values (expressed in arbitrary units, a.u.).

The fruity series increased significantly in both grape varieties, by 22.9% in CS and 26.8% in FN (*p* < 0.05), driven by higher concentrations of key esters. Isoamyl acetate increased by 25.8% in CS and 73.1% in FN, ethyl octanoate by 31.2% in CS and 24.4% in FN, and 2-phenylethyl acetate by 14.3% in CS and 26.5% in FN. Although ethyl hexanoate decreased by 63.6% in CS, its effect was masked by the high-impact esters, resulting in a net enhancement of fruity perception (Figure 4).

Green fruit and green series showed contrasting responses between the two grape varieties (*p* < 0.05). In CS, concentrations decreased significantly by 22.4% and 20.3%, whereas in FN they increased by 23.8% and 31.1%, respectively. These results suggest that the zwitterionic resin interacts differently with the wine matrix, influencing the retention or release of C6-derived alcohols and aldehydes (Table 2).

The honey series also displayed variety-dependent behavior (*p* < 0.05). It decreased in CS by 14.7% but increased in FN by 30.6%. Despite the elevated concentrations of lactones and furans in CS, the perception of honey notes may be limited due to sensory saturation, whereas in FN the increase may result from additive effects within the suboptimal concentration range.

Citrus aromas decreased in CS by 27.5% (*p* < 0.05) and remained stable in FN, with a non-significant change of 2.4%, indicating limited and variety-dependent effects on terpenoid compounds (Figure 4).

Chemical and waxy series increased significantly in both grape varieties, by 14.8% and 8.4% in CS, and by 15.2% and 21.5% in FN (*p* < 0.05). These enhancements are associated with higher alcohols, such as octanol, which increased by 55.0% in CS and 49.2% in FN, and with long-chain compounds, intensifying structural aroma and body perception.

Floral notes were moderately enhanced, by 14.9% in CS and 20.4% in FN (*p* < 0.05), reflecting the preservation of terpenes (β-citronellol, nerolidol) and the increase in norisoprenoids, such as β-damascenone.

Creamy attributes increased significantly in CS by 19.8% (*p* < 0.01) and marginally in FN by 5.9% (non-significant), suggesting that lactone liberation is influenced by the initial composition of the wine matrix.

Overall, zwitterionic resin treatment acts as a selective matrix-refining agent, consistently enhancing fruity, chemical, waxy, and floral series while modulating honey, green, and citrus aromas in a variety-dependent manner. These modifications enhance aromatic complexity and balance without compromising varietal typicity.

The molecular basis of these sensory changes is supported by volatile compound analysis. Key esters, higher alcohols, aldehydes, lactones, and norisoprenoids showed increases consistent with the observed sensory trends. Three concurrent mechanisms appear to drive these effects: (1) matrix refinement and salting-out of fermentative volatiles, (2) de-complexation from polyphenolic matrices, and (3) variety-dependent modulation of perception ratios for complex sensory categories. Together, these mechanisms reposition the aromatic balance toward enhanced fruitiness, floral complexity, and structural intensity, while precisely modulating vegetative and sweet notes based on initial wine composition.

These modifications demonstrate that zwitterionic resin treatment acts as a selective matrix-refining agent, consistently enhancing fruity, chemical, waxy, and floral series (all *p* < 0.05) while influencing honey, green, and citrus notes in a variety-dependent manner. These modifications increase aromatic complexity and balance, supporting targeted ion-exchange strategies for precision wine aroma engineering without compromising varietal typicity.

### 3.4. Multivariate Analysis of Volatile Profiles

#### 3.4.1. Principal Component Analysis (PCA)

**Figure 5:**
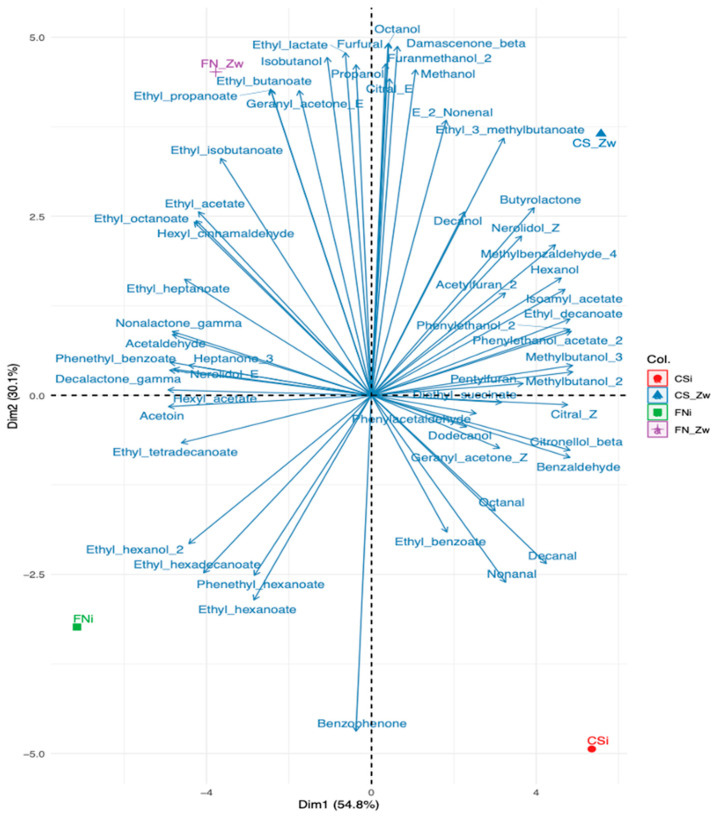
Principal component analysis (PCA) of the volatile compounds in initial Cabernet Sauvignon (CSi) and Fetească neagră (FNi) wines as compared to zwitterionic resin-treated samples (CS-Zw, FN-Zw).

Principal component analysis (PCA) was performed on all quantified volatile organic compounds across three replicate samples for each treatment and variety (CSi—initial Cabernet Sauvignon; CS-Zw—Cabernet Sauvignon treated with zwitterionic resin; FNi—initial Fetească neagră; FN-Zw—Fetească neagră treated with zwitterionic resin) to assess the impact of zwitterionic resin application on wine volatile composition. The PCA revealed clear discrimination of wine samples according to both grape variety and resin treatment. The first two principal components explained 84.9% of the total variance: PC1 (54.8%) primarily reflected varietal differences, separating FN samples (negative scores) from CS samples (positive scores), while PC2 (30.1%) reflected the treatment effect (Figure 5).

The distribution of volatile compounds within the PCA space highlighted distinct aromatic signatures for each treatment. Cabernet Sauvignon-treated wines (CS-Zw) were associated with higher levels of fruity esters and higher alcohols, including isoamyl acetate, ethyl decanoate, and hexanol, together with compounds such as furfural, octanol, and damascenone. The presence of furfural in the treated samples likely reflects de-complexation of furfural precursors during matrix refinement.

In contrast, initial CS wines (CSi) were characterized by compounds such as butyrolactone and hexanol, which contribute to the characteristic green and floral aromatic profile of the wine. For FN-treated samples (FN-Zw) were associated with higher levels of lipid-derived esters and phenolic compounds, including ethyl hexanoate and benzophenone. Meanwhile, initial samples (FNi) were linked to compounds such as nonalactone, nerolidol, and acetoin, which are typically associated with creamy and fermentation-derived aromatic notes.

Overall, the PCA results demonstrate that both grape variety and zwitterionic resin treatment influence the volatile composition of the wines. Fetească neagră samples were mainly associated with lactones and ethyl esters contributing to fruity and floral aromas, whereas Cabernet Sauvignon samples were more strongly linked to aldehydes and lipid-oxidation-derived volatiles. These differences likely reflect variety-specific metabolic pathways related to lipid oxidation and ester formation, which shape the volatile profiles of the wines, while the separation observed along PC2 indicates that the zwitterionic resin treatment selectively modulates the aromatic balance without altering varietal typicity.

#### 3.4.2. Heatmap Analysis

**Figure 6:**
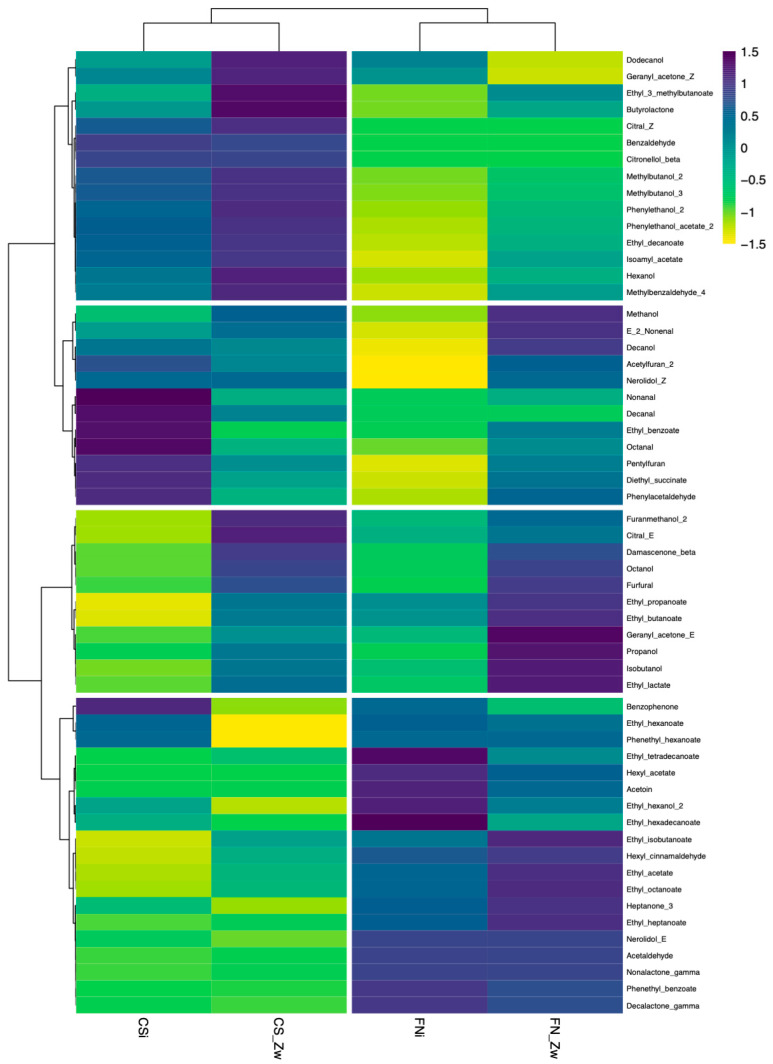
Heatmap analysis of the volatile compounds in initial Cabernet Sauvignon (CSi) and Fetească neagră (FNi) wines compared to zwitterionic resin-treated samples (CS-Zw, FN-Zw).

Hierarchical clustering of all volatile compounds revealed clear grouping by variety (CS vs. FN) and treatment (initial vs. treated), confirming PCA findings (Figure 6).

Within each variety, a secondary separation between treated and initial wines was observed, indicating that the zwitterionic resin treatment applied during the stabilization stage modified the relative abundance of several volatile compounds.

Distinct distribution patterns were observed for several compound groups. CS wines were associated with higher levels of aldehydes and oxidation-related volatiles (benzaldehyde, octanal, decanal). In contrast, FN wines showed higher levels of lactones and ethyl esters linked to fruity/floral aromas.

Notably, the treated samples showed increased relative abundance of several esters, including isoamyl acetate, ethyl decanoate, and phenylethanol, compounds known to contribute to fruity and floral aromas. This correspondence matches sensory results: fruity +22.9% CS (*p* < 0.01), +26.8% FN (*p* < 0.001); floral +14.9% CS (ns), +20.4% FN (*p* < 0.01).

In CS wines, the treatment also resulted in a reduction in compounds associated with herbaceous notes, corresponding to the observed decrease in green fruit and citrus sensory descriptors, by 22% and 28%, respectively (*p* < 0.01). This reduction may be related to lower levels of aldehydes and higher alcohols, such as hexanol and nonanal, which are typically linked to green aromatic characteristics.

Despite these compositional modifications, the clustering pattern indicates that varietal identity remained the dominant factor, while treatment represented a secondary source of variation. This observation suggests that zwitterionic resin treatment can modulate specific aroma compounds without disrupting the intrinsic aromatic typicity of the wines.

## 4. Conclusions

This study demonstrates that zwitterionic resin treatment enhances higher alcohols and esters in red wines while preserving aldehydes, ketones, and varietal markers. Heatmap analysis revealed clear varietal differences, with FN showing higher levels of esters (fruity notes) and CS exhibiting elevated aldehydes (green and lipid-derived aromas). PCA confirmed these findings, with the first two components explaining 84.9% of variance: PC1 reflected varietal differences, and PC2 captured the impact of resin treatment, producing consistent compositional changes in both wine types.

Fetească neagră wines treated with zwitterionic resin (FN-Zw) were enriched in lipid-derived esters and phenolic compounds, whereas the initial Fetească neagră (FNi) was dominated by nonalactone, nerolidol, and acetoin, contributing to creamy and fermentation-derived notes. Similarly, Cabernet Sauvignon wines treated with zwitterionic resin (CS-Zw) showed higher levels of fruity esters and alcohols, while the initial Cabernet Sauvignon (CSi) preserved its characteristic green and floral aromas.

Zwitterionic resins selectively reduced phenolic content and tannins in both CS and FN wines, enhancing colloidal stability. The treatment also improved wine clarity and lightness, especially in Fetească neagră, without compromising color integrity, offering a controlled strategy for stabilizing and visually polishing red wines.

Zwitterionic treatment preserves key physicochemical parameters while modulating aroma composition, demonstrating its potential as a multifunctional tool for enhancing wine aromatic expression and complexity. Further studies are planned to investigate the retention of heavy metals and other trace elements in wines treated with zwitterionic resins.

## Figures and Tables

**Table 1:** Volatile aroma compounds determined in wines before and after treatment with zwitterionic ion-exchange resins. Different letters (a–d) indicate statistically significant differences at a 95% confidence level (*p* < 0.05) among experimental variants.

	CSi	CS-Zw	FNi	FN-Zw
**Alcohols**				
**Major alcohols (mg/L)**	**653 ± 3 b**	**728 ± 1 a**	**490 ± 2 d**	**572 ± 4 c**
Methanol	144 ± 9 b	159 ± 3 a	137 ± 4 b	166 ± 10 a
Propanol	34.1 ± 0.9 c	38.7 ± 0.9 b	34 ± 5 b	43.2 ± 0.5 a
Isobutanol	59 ± 2 c	65 ± 3 b	61 ± 0.8 c	69.1 ± 0.5 a
2-Methylbutanol	59 ± 3 b	65.6 ± 0.7 a	35.3 ± 0.4 d	39.93 ± 0.08 c
3-Methylbutanol	300 ± 6 b	333 ± 3 a	186 ± 3 d	211 ± 3 c
2-Phenylethanol	58 ± 5 b	68 ± 2 a	36 ± 1 d	43 ± 3 c
**Minor alcohols (μg/L)**	**581 ± 42 b**	**749 ± 38 a**	**422 ± 38 c**	**533 ± 21 b**
Hexanol	483 ± 42 b	615 ± 35 a	317 ± 33 d	396 ± 18 c
2-Ethyl-1-hexanol	29 ± 1 a	27 ± 1 b	32 ± 2 a	30 ± 1 a
Octanol	60 ± 2 b	93 ± 7 a	63 ± 3 b	94 ± 9 a
Decanol	3.3 ± 0.1 b	3 ± 0.3 b	1.61 ± 0.08 c	4.1 ± 0.3 a
Dodecanol	2.2 ± 0.2 a	2.6 ± 0.2 a	2.3 ± 0.3 a	1.9 ± 0.2 b
2-Furanmethanol	4.2 ± 0.3 d	8.2 ± 0.2 a	5.2 ± 0.5 c	6.9 ± 0.5 b
**Esters**				
**Major esters (mg/L)**	**189 ± 2 d**	**210.1 ± 0.2 c**	**225 ± 2 b**	**261 ± 4 a**
Ethyl acetate	72 ± 1 d	88 ± 0.5 c	113 ± 2 b	130.7 ± 0.6 a
Ethyl lactate	94 ± 6 b	104 ± 1 a	96 ± 5 a	110 ± 9 a
Diethyl succinate	22 ± 2 a	18.5 ± 0.9 b	16 ± 2 b	20 ± 2 a
**Minor esters (μg/L)**	**1058 ± 54 b**	**1243 ± 58 a**	**724 ± 54 d**	**949 ± 25 c**
Ethyl propanoate	22.8 ± 0.9 c	33 ± 2 b	31 ± 4 b	38 ± 1 a
Ethyl isobutanoate	28 ± 3 d	44 ± 3 c	54 ± 6 b	74 ± 1 a
Ethyl butanoate	69 ± 6 c	85 ± 4 b	82 ± 8 b	94 ± 2 a
Ethyl 3-methylbutanoate	7.42 ± 0.08 c	13.3 ± 0.4 a	5.8 ± 0.4 d	8.7 ± 0.3 b
Isoamyl acetate	399 ± 12 b	502 ± 24 a	160 ± 16 d	277 ± 13 c
Ethyl hexanoate	55 ± 4 a	20 ± 2 b	57 ± 3 a	51 ± 6 a
Hexyl acetate	N.D.	N.D.	0.95 ± 0.06 a	0.64 ± 0.02 b
Ethyl heptanoate	0.01 ± 0 d	0.05 ± 0.01 c	0.39 ± 0.02 b	0.52 ± 0.01 a
Ethyl benzoate	0.16 ± 0.01 a	0.12 ± 0.01 b	0.12 ± 0.05 a	0.14 ± 0.01 a
Ethyl octanoate	32 ± 3 d	42 ± 1 c	63 ± 2 b	78.4 ± 0.9 a
2-Phenylethanol acetate	412 ± 29 b	471 ± 27 a	234 ± 20 d	296 ± 15 c
Ethyl decanoate	11.1 ± 0.7 b	13.8 ± 0.7 a	4.3 ± 0.3 d	6.8 ± 0.3 c
Phenethyl hexanoate	0.2 ± 0.01 a	0.19 ± 0 a	0.2 ± 0 a	0.2 ± 0 a
Ethyl tetradecanoate	5.2 ± 0.4 c	5.5 ± 0.6 b	8,1 ± 0.4 a	6.3 ± 0.2 b
Phenethyl benzoate	1.26 ± 0.02 b	1.25 ± 0.02 b	2.2 ± 0.2 a	2.08 ± 0.05 a
Ethyl hexadecanoate	15.2 ± 0.2 b	13.7 ± 0.9 c	22 ± 2 a	15.5 ± 0.7 b
**Aldehydes**				
**Major aldehydes (mg/L)**	**51 ± 4 b**	**53 ± 1 b**	**112.6 ± 0.9 a**	**111 ± 4 a**
Acetaldehyde	51 ± 4 b	53 ± 1 b	112.6 ± 0.9 a	111 ± 4 a
**Minor aldehydes (μg/L)**	**186 ± 6 b**	**214 ± 10 a**	**39.1 ± 0.9 d**	**69 ± 2 c**
Benzaldehyde	96 ± 6 a	80 ± 3 b	N.D.	N.D.
Octanal	1.2 ± 0.1 a	0.97 ± 0.06 b	0.9 ± 0.1 b	1.03 ± 0.09 a
Nonanal	5.5 ± 0.4 a	4 ± 0.3 b	3.7 ± 0.2 b	4 ± 0.2 b
Decanal	5.3 ± 0.5 a	3.8 ± 0.3 b	2.8 ± 0.2 c	2.8 ± 0.3 c
Phenylacetaldehyde	23 ± 3 a	18.1 ± 0.5 b	16 ± 2 b	21 ± 2 a
4-Methylbenzaldehyde	49 ± 4 b	102 ± 7 a	11.6 ± 0.1 d	33 ± 4 c
E-2-Nonenal	3.9 ± 0.3 b	4.4 ± 0.4 a	3 ± 3 a	5 ± 0.4 a
Hexyl cinnamaldehyde	1.04 ± 0.07 b	1.16 ± 0.08 b	1.3 ± 0.2 a	1.34 ± 0.08 a
**Ketones**				
**Major ketones (mg/L)**	**15 ± 2 b**	**15 ± 1 b**	**18.1 ± 0.5 a**	**17 ± 0.2 b**
Acetoin	15 ± 2 b	15 ± 1 b	18.1 ± 0.5 a	17 ± 0.2 b
**Minor ketones (μg/L)**	**5 ± 0.4 b**	**4.6 ± 0.1 b**	**5.5 ± 0.4 a**	**5.6 ± 0.1 a**
Benzophenone	1.43 ± 0.07 a	1.26 ± 0.09 b	1.38 ± 0.04 a	1.3 ± 0.2 a
3-Heptanone	3.6 ± 0.4 a	3.36 ± 0.08 b	4.1 ± 0.4 a	4.3 ± 0.3 a
**Furanic compounds (μg/L)**	**404 ± 30 b**	**509 ± 28 a**	**385 ± 28 b**	**531 ± 20 a**
Furfural	355 ± 31 b	473 ± 27 a	361 ± 28 b	489 ± 22 a
2-Acetylfuran	36 ± 3 a	32 ± 1 a	23.6 ± 0.7 b	35 ± 2 a
Pentylfuran	13 ± 1 a	4.7 ± 0.2 b	0.69 ± 0.03 c	6 ± 2 b
**Lactones (μg/L)**	**35,943 ± 2864 b**	**49,219 ± 1734 a**	**29,273 ± 2049 c**	**34,706 ± 2841 b**
Butyrolactone	35,923 ± 2862 b	49,198 ± 1735 a	29,216 ± 2049 c	34,652 ± 2841 b
γ-Nonalactone	19 ± 2 b	20.3 ± 0.9 b	51 ± 5 a	50 ± 3 a
γ-Decalactone	1.5 ± 0.2 b	1.4 ± 0.1 b	5.4 ± 0.6 a	4.7 ± 0.2 a
**Terpenes and norisoprenoids (μg/L)**	**45 ± 3 a**	**46.7 ± 0.8 a**	**7.1 ± 0.2 c**	**8 ± 0.2 b**
B-Citronellol	36 ± 3 a	35.9 ± 0.9 a	N.D.	N.D.
Z-Citral	1.6 ± 0.1 b	2.4 ± 0.2 a	N.D.	N.D.
E-Citral	0.02 ± 0.01 d	0.7 ± 0.03 a	0.21 ± 0.01 c	0.42 ± 0.04 b
E-Nerolidol	2.62 ± 0.02 a	2.61 ± 0.01 b	2.68 ± 0.05 a	2.68 ± 0.05 a
Z-Nerolidol	0.09 ± 0.01 a	0.09 ± 0.01 a	0.02 ± 0 b	0.09 ± 0.01 a
B-Damascenone	2.1 ± 0.2 b	3 ± 0.2 a	2.2 ± 0.2 b	2.9 ± 0.2 a
E-Geranyl Acetone	0.43 ± 0.02 a	0.45 ± 0.02 a	0.44 ± 0.03 a	0.48 ± 0.06 a
Z-Geranyl Acetone	1.57 ± 0.08 a	1.7 ± 0.2 a	1.55 ± 0.04 a	1.4 ± 0.1 a

CSi—initial Cabernet Sauvignon; CS-Zw—Cabernet Sauvignon treated with zwitterionic resin; FNi—initial Fetească neagră; FN-Zw—Fetească neagră treated with zwitterionic resin. N.D.—not detected.

**Table 2:** Aroma series of wines treated with zwitterionic resin treatment.

	CSi	CS-Zw	ΔCS (%)	FNi	FN-Zw	ΔFN (%)
Fruity	35 ± 2	43 ± 2	+22.9	38 ± 2	48.2 ± 0.5	+26.8
Green fruit	8.5 ± 0.5	6.6 ± 0.3	−22.4	6.3 ± 0.4	7.8 ± 0.8	+23.8
Green	6.4 ± 0.7	5.1 ± 0.1	−20.3	4.5 ± 0.4	5.9 ± 0.6	+31.1
Creamy	2.42 ± 0.11	2.9 ± 0	+19.8	3.4 ± 0.2	3.6 ± 0.1	+5.9
Citrus	6.9 ± 0.6	5 ± 0.4	−27.5	4.1 ± 0.2	4.2 ± 0.4	+2.4
Chemistry	26.4 ± 0.3	30.3 ± 0.2	+14.8	27.7 ± 0.3	31.9 ± 0.3	+15.2
Honey	7.5 ± 0.6	6.4 ± 0.2	−14.7	4.9 ± 0.4	6.4 ± 0.5	+30.6
Waxy	10.7 ± 0.2	11.6 ± 0.4	+8.4	14.9 ± 0.1	18.1 ± 0.4	+21.5
Floral	8.7 ± 0.3	10 ± 0.3	+14.9	4.9 ± 0.2	5.9 ± 0.3	+20.4

CSi—initial Cabernet Sauvignon; CS-Zw—Cabernet Sauvignon treated with zwitterionic resin; FNi—initial Fetească neagră; FN-Zw—Fetească neagră treated with zwitterionic resin.

## Data Availability

The original contributions presented in this study are included in the article. Further inquiries can be directed to the corresponding authors.
